# Supplementary figures and images for: In vivo analysis reveals that ATP-hydrolysis couples remodeling to SWI/SNF release from chromatin
Source: eLife. 2021 Jul 27;10:e69424. doi: 10.7554/eLife.69424 (PMC8352592; doi:10.7554/eLife.69424)

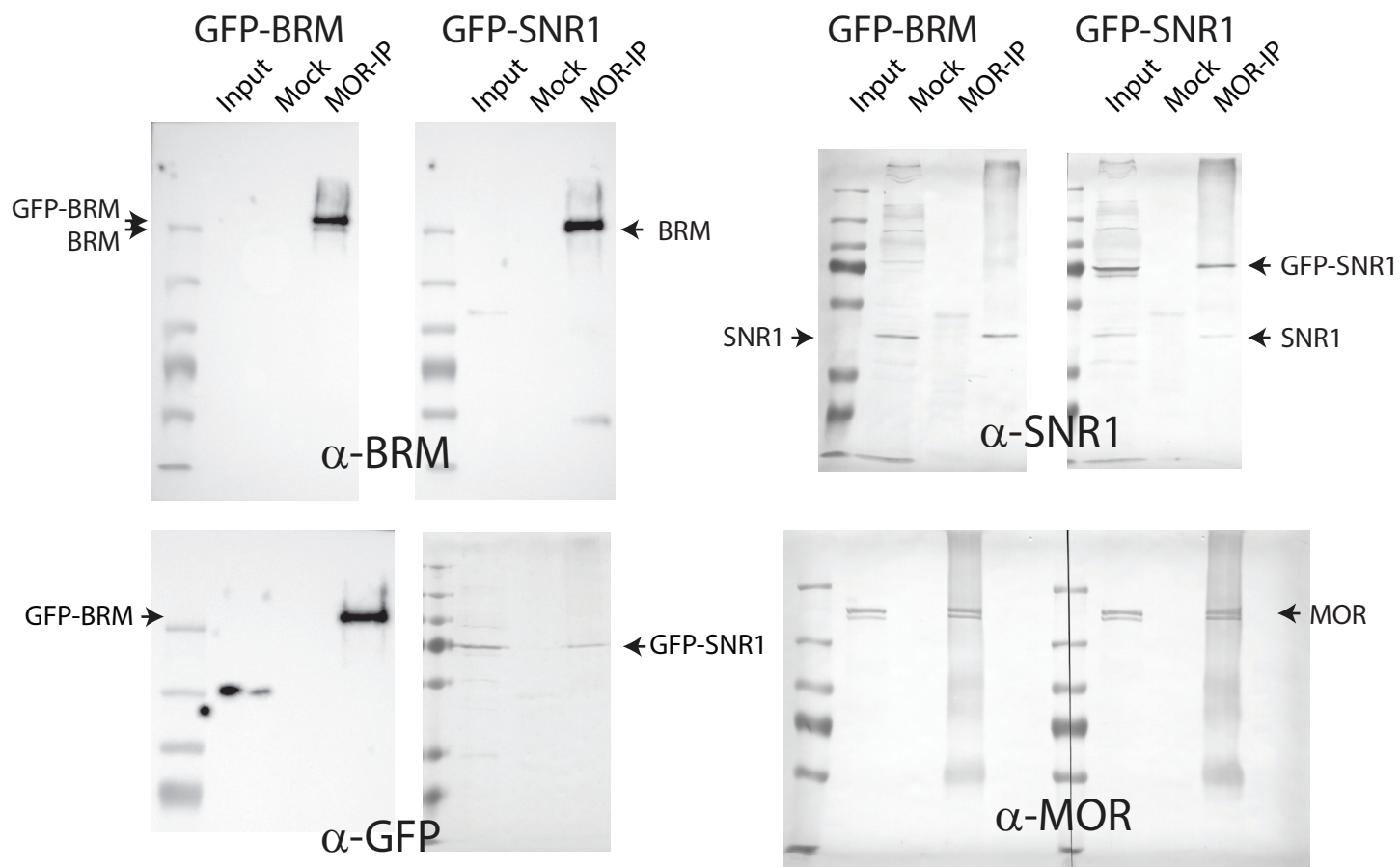

Supplement: Figure 1—figure supplement 1—source data 1. [file elife-69424-fig1-figsupp1-data1.pdf]

Figure 4A, source data, original blots

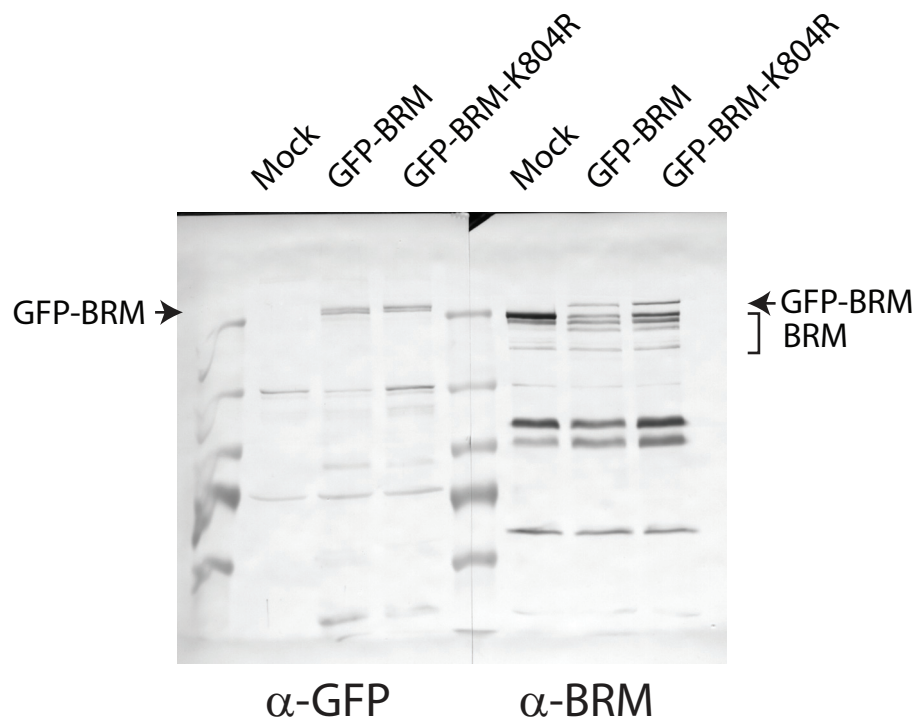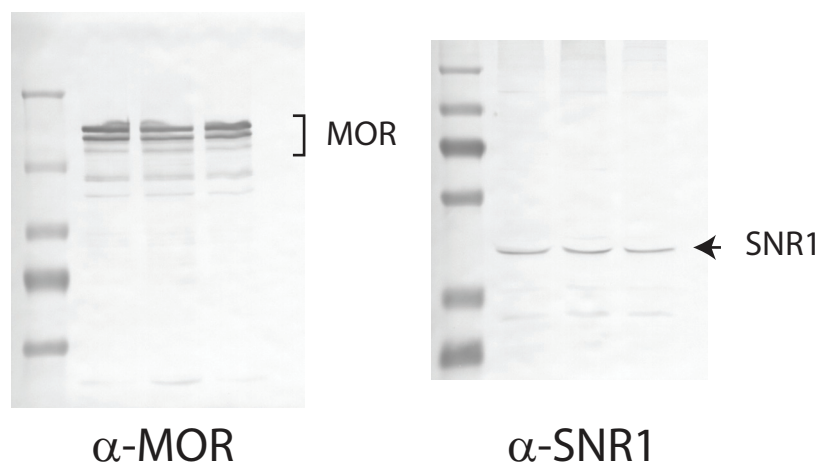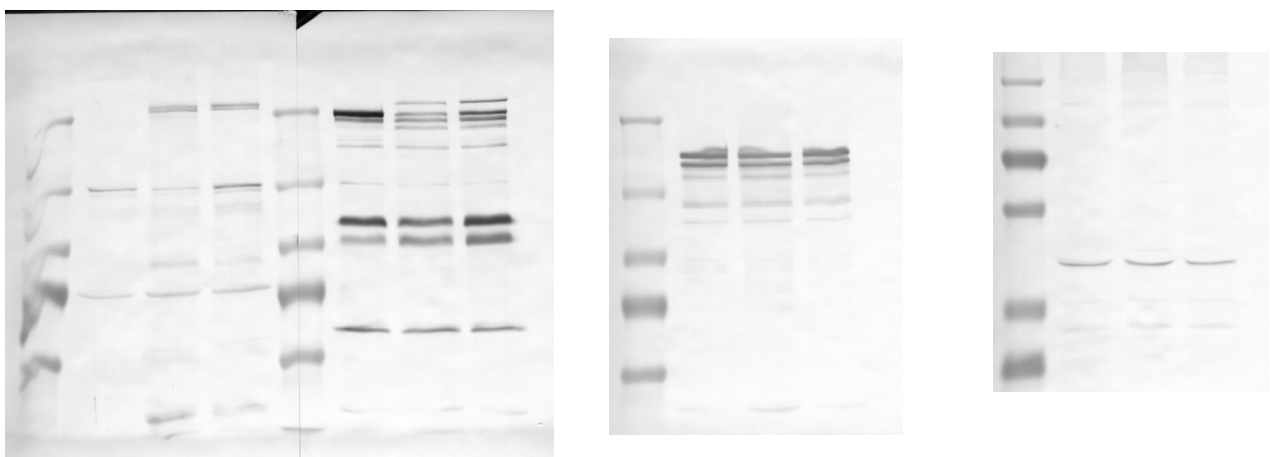

Supplement: Figure 4—source data 1. [file elife-69424-fig4-data1.pdf]
