# Supplementary material for: In vivo analysis reveals that ATP-hydrolysis couples remodeling to SWI/SNF release from chromatin
Source: eLife. 2021 Jul 27;10:e69424. doi: 10.7554/eLife.69424 (PMC8352592; doi:10.7554/eLife.69424)
Supplement: Supplementary file 1. [file elife-69424-supp1.docx]

**Supplementary Table 1. List of *Drosophila* lines**

| **Name** | **Insertion** | **Source, identifier** |
| --- | --- | --- |
| GFP-BRD7 | yw; pTG-BRD7 / cyo | PV-lab, PV136 |
| GFP-BRM | yw; pTG-BRM / cyo | PV-lab, 13587-1-5 |
| GFP-BRM_low_ | Mi{PT-GFSTF.1}brm[MI01941-GFSTF.1] | PV-lab, 59784 |
| GFP-BRM-K804R | yw; pTG-BRM K804R / cyo | PV-lab, 16460-2-2/9 |
| mCh-BRM-K804R | yw; pTCh-BRM K804R / cyo | PV-lab, 16460-1-1/9 |
| GFP-D4 | yw; pTG-D4 / cyo | PV-lab, PV144 |
| GFP-SNR1 | yw; pTG-SNR1 / cyo | PV-lab, PV138 |
| GFP-EcR | W[1118]; pTG-EcR / cyo | PV-lab, 26183-1-2/6 |
| mCh-EcR | W[1118]; pTCh-EcR / cyo | PV-lab, 26183-3-6/10 |
| GFP-H2A | Act5C-Gal4 – UAS-GFP-H2A / cyo | PV-lab, Act-GFP-H2A-2131 |
| GFP-H2B | Act5C-Gal4 – UAS-GFP-H2B / cyo | PV-lab, Act-GFP-H2B-2144 |
| mCh-H2B | Sgs3-Gal4 – UAS-GFP-H2B / Tm6b | PV-lab, SgPV100 |
| mCh-HP1 | UAS-mCh-HP1, 2^nd^ | Gift from J-M Gilbert and F. Karch (Geneva), UAS0004204 |
| GFP-PC |  | Gift from R Paro (Dietzel et al, 1999) |
| GFP-RPB2 | yw; pTG-RPB2 / TM3 | PV-lab, PV3 |
| mCh-RPB3 | yw; pTCh-RPB3 / TM3 | PV-lab, PV65 |
| NLS-GFP | UAS-NLS-NES^P12^-GFP | Bloomington, 70330 |
| MOR^KD^ |  | VDRC, 110712 |
| Act5C-GAL4 | P{Act5C-Gal4} | Bloomington, 4414 |
| Sgs3-GAL4 | P{Sgs3-GAL4.PD} | Bloomington, 6870 |
