# Supplementary material for: In vivo analysis reveals that ATP-hydrolysis couples remodeling to SWI/SNF release from chromatin
Source: eLife. 2021 Jul 27;10:e69424. doi: 10.7554/eLife.69424 (PMC8352592; doi:10.7554/eLife.69424)
Supplement: Supplementary file 2. [file elife-69424-supp2.docx]

**Supplementary Table 2. List of Primers**

| **Assay** | **target** | **identifier** | **Sequence** |
| --- | --- | --- | --- |
| **ChIP-qPCR** | E74B | PV3537 | TGCGTCTTCCTCTTCACC |
|  |  | PV3538 | AGAACCCCACGAAATCCC |
|  | E74A | PV3531 | ACGCTCAAGTTCACGCTCTG |
|  |  | PV3532 | TCCCTACTCTCTTTGGCTCTCC |
|  | Antp | PV10018 | AGATGCAGCGATGGCAGATA |
|  |  | PV10019 | ATGTTGCGGGGGATAAGGTC |
|  | IAB9-PRE | PV10030 | TCACCTCAGCCGGCCATAAT |
|  |  | PV10031 | CTCACTTGACGATCGCCGTA |
|  | glcAT-P | PV10570 | CCAGCTGATAGCCAACCCTT |
|  |  | PV10571 | AGCCAAGACCTCCTAACCCT |
|  | tna | PV10576 | CTGTCTCTGTCGCACTGCAT |
|  |  | PV10577 | CGTCGGTCAGTCGGCAAATA |
|  | Vsx1-P | PV10137 | GGGTTGGACGATAACCCCTC |
|  |  | PV10138 | AAACAACTTGCAGCGCCATC |
|  | Vsx1-gene | PV9658 | GAATACGGATTGTATGGAGC |
|  |  | PV9659 | GTAGCTGGTGTTCGTATCGT |
|  | CG1998 | PV10560 | ACACGGCGAGACACCAATAC |
|  |  | PV10561 | AGCCTAGGTACTCATCCCGA |
|  | -2k (bxd) | PV3299 | ATCTACGATATTGCCTTTGCC |
|  |  | PV3300 | CCTTCTCAAGACAACTTCTGG |
|  | bxd-PRE | PV3277 | CTCCCTCTCTCCGCAGTC |
|  |  | PV3278 | AACCATTCAGATTCAGCAAACG |
| **RT-qPRC** | E74 common | PV3094 | CCGCTGCCCGAGGACAAG |
|  |  | PV3095 | CAATTAGCCCAAGCACAGACACC |
|  | E75 common | PV8191 | GAACGGAGCCAATGCCCGCT |
|  |  | PV8192 | CAGGCAGCCCTTGAGTCGCG |
|  | CG11874 | PV2953 | AGTGTTGCTCTGCCTAAGTGG |
|  |  | PV2954 | CGGATGATGGTGCGGATTGG |
