# Supplementary material for: In vivo analysis reveals that ATP-hydrolysis couples remodeling to SWI/SNF release from chromatin
Source: eLife. 2021 Jul 27;10:e69424. doi: 10.7554/eLife.69424 (PMC8352592; doi:10.7554/eLife.69424)
Supplement: Supplementary file 3. [file elife-69424-supp3.docx]

**Supplementary Table 3. List of Antibodies**

| **Target** | **Assay** | **Source, identifier** |
| --- | --- | --- |
| α-Brm: rabbit polyclonal antibody raised against peptide C-QTRRKRSQKKYTISDD | WB | PV-lab, GR987 and GR988 |
| α-Brm rabbit polyclonal antibody (affinity purified) | Polytene & Whole mount IF | PV-lab, PV21-PV22,  Kal et al., 2000 |
| α-histone guinea pig polyclonal antibody raised against purified *Drosophila* core histones | Polytene IF | PV-lab, GR764 |
| α-EcR mouse monoclonal antibody | Polytene IF | DSHB, DDA2.7 (EcR common) deposited by Thummel & Hogness |
| α-EcR mouse monoclonal antibody | Polytene IF | DSHB, Ag10.2 (EcR common) deposited by Thummel & Hogness |
| α-GFP guinea pig polyclonal antibody raised against GFP-GST fusion protein | WB | PV-lab, GR955 |
| α-GFP guinea pig polyclonal antibody raised against GFP-GST fusion protein | Polytene IF | PV-lab, GR956 |
| α-GFP | WB & Polytene IF | SicGen, AB0020-500 |
| α-MOR rabbit polyclonal antibody | WB, Polytene & Whole mount IF | PV-lab, PV127, Mohrmann et al., 2004 |
| α-RNA polymerase II | Whole mount IF | Covance, Mix of H5-MMS-129R; 8WG16 and H14-MMS-134R |
| α-RNA polymerase IIo^ser2^ | Polytene | Covance, H5-MMS-129R |
| α-PBRM rabbit polyclonal antibody | Polytene | PV-lab, GR51, Mohrmann et al., 2004 |
| α-OSA, mouse monoclonal antibody | Polytene IF | Treisman et al., 1997 |
| α-SNR1 guinea pig polyclonal antibody | WB & Whole mount IF | PV-lab, GR150, Chalkley et al., 2008 |
| Alexa-Fluor secondary antibodies | Polytene & Whole mount IF | Thermo-Fisher, A-11012, A-11073, A-11029, A-11032, A-11008, A11055, A-21207 |

**References**

Chalkley, G.E., Moshkin, Y.M., Langenberg, K., Bezstarosti, K., Blastyak, A., Gyurkovics, H., Demmers, J.A. and Verrijzer C.P. (2008). The transcriptional coactivator SAYP is a trithorax group signature subunit of the PBAP chromatin remodeling complex. Mol. Cell Biol. 28, 2920-2929. <https://doi.org/10.1128/MCB.02217-07>.

Mohrmann, L., Langenberg, K., Krijgsveld, J., Kal, A.J., Heck, A.J. and Verrijzer, C.P. (2004). Differential targeting of two distinct SWI/SNF-related Drosophila chromatin-remodeling complexes. Mol. Cell. Biol. 27, 651-661. <https://doi.org/10.1128/mcb.24.8.3077-3088.2004>.

Treisman, J. E., A. Luk, G. M. Rubin, and U. Heberlein. (1997). Eyelid antagonizes wingless signaling during Drosophila development and has homology to the Bright family of DNA-binding proteins. Genes Dev.11,1949-1962. <https://doi.org/10.1101/gad.11.15.1949>.
